# Sex Hormones and Gender Role Relate to Gray Matter Volumes in Sexually Dimorphic Brain Areas

**DOI:** 10.3389/fnins.2019.00592

**Published:** 2019-06-18

**Authors:** Belinda Pletzer

**Affiliations:** ^1^Department of Psychology, University of Salzburg, Salzburg, Austria; ^2^Centre for Cognitive Neuroscience, University of Salzburg, Salzburg, Austria

**Keywords:** sex hormones, gender role, brain structure, oral hormonal contraceptives, sex differences

## Abstract

The present study investigates the relationship of circulating sex hormone levels and gender role to gray matter volumes in sexually dimorphic brain areas and explores, whether these relationships are modulated by biological sex (as assigned at birth based on sexual anatomy) or oral contraceptive (OC) use. It was hypothesized that testosterone and masculinity relate positively to gray matter volumes in areas that are typically larger in men, like the hippocampus or cerebellum, while estradiol/progesterone and femininity relate positively to gray matter volumes in the frontal cortex. To that end, high resolution structural MRI scans, sex hormone levels and gender role self-assessments were obtained in a large sample 89 men, 89 naturally cycling (NC) women, and 60 OC users. Men showed larger regional gray matter volumes than women in the cerebellum and bilateral clusters spanning the putamen and parts of the hippocampi/parahippocampi and fusiform gyri. In accordance with our hypotheses, a significant positive association of testosterone to hippocampal volumes was observed in women irrespective of OC use. Participant’s self-reported femininity was significantly positively associated with gray matter volumes in the left middle frontal gyrus (MFG) in men. In addition several differences between OC-users and NC women were identified.

## Introduction

Sex differences in brain structure and function have long been a matter of debate and have attracted considerable research interest, as they are assumed to underlie sex differences in behavior (e.g., Cosgrove et al., 2007; Andreano and Cahill, 2009). For instance, sex differences in the brain are thought to explain sex differences in cognition, or the differential vulnerability for neurodevelopmental and psychiatric disorders in men and women.

Sex differences in brain structure have repeatedly been reported, with consistencies in some areas, but inconsistencies in others (see Ruigrok et al., 2014 for a meta-analysis). Regional gray matter volumes change with age at a different rate in males and females, both during development (Gur and Gur, 2016) and during aging (Jäncke et al., 2015). These age-related changes account for some of the variability between studies. Recent meta-analyses (Ruigrok et al., 2014) and more large-scale studies (Ritchie et al., 2018) arrive at similar conclusions. In adults, larger regional volumes in males compared to females are consistently reported in subcortical areas, including the hippocampus, amygdala, basal ganglia and nucleus accumbens, in parts of the parahippocampal gyrus, in the cerebellum and the posterior cingulate cortex (PCC). Larger regional volumes in females compared to males are consistently reported in frontal areas, including the anterior cingulate cortex (ACC).

It can be speculated whether males’ larger hippocampal/parahippocampal volumes play a role in their frequently reported advantage in spatial tasks (Andreano and Cahill, 2009; Levine et al., 2016) or whether females’ larger volumes in frontal areas play a role in their frequently reported advantage in verbal tasks (Andreano and Cahill, 2009) or self-control (e.g., Chapple et al., 2010; Hosseini-Kamkar and Morton, 2014). However, the relationship between structure and function is not always as clear-cut, and the larger volume of a brain area doesn’t necessarily lead to better performance in tasks involving this area. As a counter-example, the hippocampus has also been implicated in verbal memory tasks and larger hippocampal volumes relate to better verbal memory performance in females (Protopopescu et al., 2008). Also in females, larger volumes of the fusiform face area relate to better performance in a face-recognition task (Pletzer et al., 2015a). Nevertheless, these areas show regionally larger volumes in men (e.g., Pletzer et al., 2010), while women outperform men in verbal memory and face-recognition tasks (see Andreano and Cahill, 2009 for a review).

Sex differences in brain morphology are thought to result from organizational effects of sex hormones on the brain during development – both prenatally and during adolescence (Kelly et al., 1999; Cosgrove et al., 2007). At a smaller scale, sex hormones appear to also exert activational effects on the brain throughout our adult life span, the most prominent example being menstrual cycle-dependent changes (Protopopescu et al., 2008; Lisofsky et al., 2015; Barth et al., 2016; Pletzer et al., 2018). The hippocampus has consistently been reported to increase gray matter volumes during the high estradiol pre-ovulatory phase (Protopopescu et al., 2008; Lisofsky et al., 2015; Barth et al., 2016; Pletzer et al., 2018), while an increase in right basal ganglia volumes has been observed in the high progesterone luteal phase (Protopopescu et al., 2008; Pletzer et al., 2018). In good accordance, animal studies also report increases in hippocampal spine density in response to estradiol (Woolley and McEwen, 1993, 1994). Furthermore, animal studies have also found similar estradiol-dependent changes in spine-density in the frontal cortex (e.g., Hao et al., 2006). These changes are more subtle and short-lived, but suggest that sex hormones continuously reshape our brain, particularly in areas, that are rich in sex hormone receptors (Barth et al., 2015). Apart from brain areas involved in the regulation of neuroendocrine axes (i.e., hypothalamus), areas with a particularly high density of sex hormone receptors include the hippocampus, the frontal cortex and the cerebellum (Barth et al., 2015). These are the same areas that show the strongest sexual dimorphism (Cosgrove et al., 2007; Ruigrok et al., 2014). Nevertheless, only few studies have addressed whether circulating levels of sex hormones relate to gray matter volumes in these areas across participants. For instance it has been demonstrated, that cross-sex hormone treatment in male-to-female and female-to-male transsexuals alters their brain structure toward the proportions of the aspired sex (Hulshoff Pol et al., 2006; Guillamon et al., 2016). However, it has not been addressed whether subjects with higher circulating testosterone levels also display larger volumes in brain areas known to be larger in men, and whether vice versa, subjects with higher circulating estradiol levels, display larger volumes in brain areas known to be larger in women. Furthermore, it is unclear, how this association may be modulated by the biological sex of participants.

Due to the accumulating knowledge of sex hormone actions on the human brain and age-related changes therein, sex hormones have been implicated as one potential cause for sex differences in cognitive functions (e.g., Kelly et al., 1999). Particularly regarding spatial abilities, a role of testosterone has been repeatedly discussed (Hooven et al., 2004; Driscoll et al., 2005; Hausmann et al., 2009; Courvoisier et al., 2013). However, most contemporary models of sex differences in cognitive functions follow a psychobiosocial approach and consider not only biological factors, like sex hormones, but also socialization (e.g., Hausmann et al., 2009; Levine et al., 2016). One social factor that has been implicated in sex differences, is gender role. Gender role refers to the predominant views of what’s typically male or typically female in a given society (e.g., Eagly and Koenig, 2006). The extent to which a person identifies with typically male characteristics, is referred to as masculinity. The extent to which a person identifies with typically female characteristics, is referred to as femininity. Unlike biological sex, gender roles are neither dichotomous nor mutually exclusive. Rather masculinity and femininity are assessed on two different continuous scales (e.g., Bem, 1974). While typical males score high on masculinity and low on femininity and typical females vice versa, a substantial proportion of individuals show no such gender-typical identification (Bem, 1974). About 30% of individuals score high on both masculinity and femininity, and are considered androgynous (e.g., Vafaei et al., 2016).

According to the gender role mediation hypothesis (Nash, 1979), the extent to which a person identifies with the societal expectations toward their biological sex transfers to their behavior, and may explain sex differences in a variety of domains, including cognitive abilities. In line with this hypothesis, a recent meta-analysis found for instance, that higher masculinity relates to better spatial performance in both men and women (Reilly and Neumann, 2013). However, while the actions of sex hormones on cognitive functions are thought to result from their actions on the brain, the relationship of gender role to brain structure and function has hardly been addressed. Only a handful of studies have assessed brain structure in untreated transgendered individuals (see Guillamon et al., 2016 for a review) and homosexuals (Ponseti et al., 2007; Abé et al., 2014; Manzouri and Savic, 2018), both groups that usually show low gender role conformity. In general, these studies suggest little differences between untreated transsexuals or homosexual and non-transsexual heterosexuals. However, these studies are characterized by small sample sizes and a certain variability in the inclusion criteria for the transsexual or homosexual groups. Thus, it remains unclear whether participants with higher masculinity show a more male-typical brain morphology, i.e., larger gray matter volumes in brain areas that are typically larger in men. Vice versa, it has not been assessed whether participants with high femininity show a more female-typical brain morphology, i.e., larger gray matter volumes in brain areas that are typically larger in women. However, a few findings do stand out. For instance, Luders et al. (2012) report larger cortical thickness in the left MFG of untreated male-to-female transsexuals compared to non-transsexual men. Abé et al. (2014) report smaller hippocampal volumes in homosexual men compared to heterosexual men. It can thus be speculated that, at least in men, gender identity is reflected to some extent in brain morphology. For instance, brain structure has been related to personality (e.g., Riccelli et al., 2017), and our understanding of what’s masculine and what’s feminine relies to a great extent on personality traits (Bem, 1974; Gruber et al., in press). Accordingly, most measures assessing masculinity and femininity include personality dimensions, like expressivity on the femininity scale and assertiveness on the masculinity scale (e.g., Eagly and Koenig, 2006). It is thus possible, that a person’s perception of how masculine or feminine they are, depends in part on their brain morphology and chemistry. A recent fMRI study has assessed brain activation in men and women during the processing of gender-related attributes (Hornung et al., 2019), as are used to assess gender role (Gruber et al., in press). They found stronger activation for gender-congruent attributes in the amygdala and putamen (Hornung et al., 2019). The present study focuses on brain morphology.

The aim of the present study is to assess whether circulating levels of sex hormones and/or participants masculinity and femininity relate to gray matter volumes in brain areas that show a high density of sex hormone receptors and have been described as sexually dimorphic. To address these questions, a hypothesis-driven region-of interest (ROI) based approach is combined with more exploratory whole-brain analyses. The hippocampus was selected as a brain area with high density of sex hormone receptors that is typically larger in men. The middle frontal gyrus (MFG) was selected as a brain area with high density of sex hormone receptors that is typically larger in women. To that end, high-resolution structural MRIs, saliva samples and gender role ratings were obtained from a large sample of 89 men and 149 women. It was hypothesized, that testosterone and masculinity relate positively to gray matter volumes in areas that are typically larger in men, like the hippocampus or cerebellum, while estradiol/progesterone and femininity relate positively to gray matter volumes in the frontal cortex. It was also explored whether these relationships are modulated by biological sex.

An important factor to consider, when addressing these questions, is hormonal contraceptive use in women. Oral hormonal contraceptives (OC) contain synthetic estrogens and progestins that do not only influence a woman’s endogenous hormonal milieu (e.g., Wiegratz et al., 2003). Previous work has outlined potential OC-dependent effects on gray matter volumes in sexually dimorphic brain areas (Pletzer et al., 2010, 2015a; De Bondt et al., 2013; Petersen et al., 2015) and on gender role (Pletzer et al., 2015b). Across different cultures, OC-users describe themselves as more feminine compared to non-users (Pletzer et al., 2015b), even though several studies indicate that their behaviors and brain activation patterns may in fact be more comparable to men (e.g., Nielsen et al., 2011; Pletzer et al., 2014). Accordingly, effects of OC-use will also be assessed in all analyses.

## Materials and Methods

### Participants

As add-on to three different neuroimaging studies, 89 men (mean age: 24.18 ± 4.44 years), 89 naturally cycling women (mean age: 24.02 ± 3.94 years), and 60 women using oral hormonal contraceptives (OC; mean age: 21.42 ± 2.46 years) completed self-ratings for their masculinity/femininity.

In all three studies, participants were right-handed, Caucasian, aged between 18 and 35 years, heterosexual, had no diagnosis of psychological, neurological or endocrinological disorders and no brain tissue abnormalities on the structural MRI. The majority of participants were university students who had completed general qualification for university entrance. All naturally cycling women had a regular menstrual cycle of 21 to 35 days length (mean duration: 29.36 ± 2.91 days).

Among them a subsample of 54 men (mean age: 24.33 ± 4.37 years) and 51 naturally cycling women (mean age: 24.12 ± 4.26 years) also completed a standardized gender role questionnaire. For those 51 women, mean cycle duration was 29.11 days (*SD* = 3.05).

Naturally cycling women and men did not differ in age (both |*t*| < 0.26; both *p* > 0.79), but hormonal contraceptive users were significantly younger than the other two groups (both *t* > 4.87, both *p* < 0.001).

### Ethics Statement

The studies were approved by the University of Salzburg’s ethics committee and conform to the Code of Ethics of the World Medical Association (Declaration of Helsinki). Informed written consent was obtained from all participants.

### Procedure

Study 1 investigated brain responses to different risk taking tasks (Pletzer and Ortner, 2016). Study 2 investigated sex differences in brain responses to numerical and attention tasks (Pletzer, 2016; Pletzer and Harris, 2018) and included only naturally cycling women. Study 3 was a resting state study (currently unpublished).

In all three studies, participants gave one saliva sample before entering the scanner and one saliva sample after scanning, both via the passive drool method. Questionnaires were completed on site immediately after the scanning session as to not interfere with the main research question of the studies. Self-ratings were included in all three studies, the GERAS was completed by participants of Study 2 and some participants of Study 1.

Among the 89 naturally cycling women in the whole sample, 58 were scanned in their luteal cycle phase (11-3 days before the onset of the next menses are counted backwards; mean cycle day: 22.03 ± 3.98). The remaining 31 women were unavailable during their luteal cycle phase and scanning sessions were scheduled during or shortly after the next menses (mean cycle day: 7.25 ± 4.07). Among the 60 hormonal contraceptive users in the whole sample, 39 used contraceptives containing androgenic progestins (Levonorgestrel, Desogestrel, Dienogest, Gestoden), while 16 used contraceptives containing anti-androgenic progestins (Drospirenon, Cyproteronacetat, Chlormadinonacetat). Five women were unable to provide information about the hormonal contraceptives they were using.

As expected, progesterone [*t*_(84)_ = 6.78, *p* < 0.001] significantly higher during the luteal cycle phase compared to the early follicular cycle phase, while testosterone and estradiol did not differ between cycle phases (both *t* < 1.29, both *p* > 0.20). However, in accordance with our previous studies hippocampal volumes did not differ significantly between menses and luteal cycle phase (both *t* < 0.50, both *p* > 0.61; compare Pletzer et al., 2018), MFG volumes were only by trend higher in the luteal cycle phase (both *t* < 1.91, both *p* = 0.06; compare Pletzer et al., 2018) and masculinity/femininity ratings did not differ significantly between menses and luteal cycle phase (all |*t*| < 1.54, all *p* > 0.13; compare Pletzer et al., 2015a). Furthermore, there were no differences between pill-types in masculinity/femininity self-ratings, sex hormones or GM-volumes (all |*t*| < 1.58, all *p* > 0.13). Accordingly, NC-women and OC-users were not split into sub-groups for the analyses.

Among the 51 naturally cycling women in the subsample, 41 were scanned in their luteal cycle phase (mean cycle day: 21.78 ± 3.85), while 10 were scanned in their menses (mean cycle day: 8.80 ± 4.60). Again, the NC group was not split by cycle phase due to the small number of participants in their menses.

### Hormone Analysis

Prior to analysis, saliva samples were stored frozen at −20° and centrifuged twice at 3000 rpm for 15 min and 10 min, respectively. As recommended by the ELISA kit instructions, aliquots from both samples were then pooled to account for fluctuation in hormone release and saliva production and obtain a more stable measure of hormone levels throughout the scanning session. Estradiol, progesterone and testosterone levels were assessed using DeMediTec^1^ salivary ELISA kits (DES6644, DES6633, and DES6622). All samples were assessed in duplicates and assessment was repeated for samples showing coefficients of variation (CV) above 25%. For estradiol, sensitivity is 1.4 pg/ml, intra-assay CV is 8.5%, inter-assay CV is 7%. For progesterone, sensitivity is 5 pg/ml, intra-assay CV is 7%, inter-assay CV is 9%. For testosterone, sensitivity is 2.2 pg/ml, intra-assay CV is 7.5%, inter-assay CV is 9%. For three participants (two men, one OC), hormone levels were not assessed due to visible blood contamination. Hormone levels of more than three *SD* above the group mean were discarded prior to analyses (E: two men, one OC, two NC; P: two OC, two NC).

### Questionnaires

#### Gender Role Self-Assessment

On a nine-point Likert-Scale, participants were asked to rate how masculine or feminine they perceived themselves in comparison to (other) men, (other) women, or the general population. The same scale was already employed by Pletzer et al. (2015b). The three comparisons were performed to take into account the fact that women tend to compare themselves to other women, while men tend to compare themselves to other men (Pletzer et al., 2015b). These ratings represent subjective measures of masculinity and femininity and depend on the participant’s personal understanding of these concepts. As outlined by Pletzer et al. (2015b), the concepts of masculinity and femininity vary between cultures and possibly also subcultures, e.g., depending on education or generation.

#### Gender-Related Attributes Scale (GERAS)

To additionally obtain a more objective measure of masculinity and femininity, a subsample of participants also performed the gender related attributes scale (GERAS). The GERAS was developed by Gruber et al. (in press) as a standardized measure to assess gender role via attributes that are typically perceived as masculine or feminine in middle European cultures. It has been well-validated and shows excellent internal consistency and reliability (Gruber et al., in press). It extends previous sex/gender role inventories (e.g., Bem Sex Role Inventory – Bem, 1981; Personal Attributes Questionnaire – Spence et al., 1975) by including not only personality traits, but also cognitive abilities and interests that are typically associated with the male or female gender on three subscales: (i) personality subscale, (ii) cognitions subscale – 14 items (7 masculine, 7 feminine), and (iii) interests subscale – 16 items (8 masculine, 8 feminine). The personality subscale consists of 20 traits (both positive and negative), 10 of which are typically associated with the male (e.g., dominant, bold) and 10 with the female gender (e.g., warm-hearted, sensitive). Participants are asked to rate how often they think these traits apply to them. The cognition subscale consists of 14 cognitive skills (7 masculine, 7 feminine), for which previous studies have demonstrated sex differences favoring men (e.g., find a way) or women (e.g., find the right words). Participants are asked to rate how well they think they are able to perform these tasks. The interests subscale consists of 16 activities, 8 of which are stereotypically preferred by men (e.g., boxing, drinking), the other 8 are stereotypically preferred by women (e.g., dancing, talking). Participants are asked to rate how interested they would be to engage in these activities. All ratings are performed on a seven-point Likert-scale. For each subscale, separate masculinity and femininity scores are obtained by averaging the ratings for masculine and feminine items, respectively. The overall masculinity and femininity scores are obtained by averaging the masculinity and femininity scores of the three subscales.

### MRI Data Acquisition and Analysis

All three studies were performed on the same scanner (Siemens Magnetom Trio Tim 3 Tesla) at the Christian Doppler Klinik (Salzburg, Austria). All studies included the same scanning sequence to obtain a high resolution structural scan using a T1-weighted sagittal 3D MPRAGE sequence (TR = 2300 ms, TE = 2.91 ms, TI delay of 900 ms, FOV 256 mm, slice thickness = 1.00 mm, flip angle 9°, voxel size 1.0 × 1.0 × 1.0 mm, 160 sagittal slices). Images were segmented into gray matter, white matter and csf partitions using cat12 standard procedures and templates. SPM12 tissue probability maps and European brain templates for affine regularization were used during the initial SPM12 affine registration, light affine preprocessing and moderate (0.5) strength of local adaptive segmentation, skull stripping and final clean-up for CAT12 segmentation. Images were spatially normalized to the same stereotactic space (MNI template) and voxel size for normalized images was set to 1.5 mm. To control for individual differences in brain size, brain segments were modulated using non-linear normalization parameters.

For ROI-based analyses, gray matter volumes were extracted from the left and right hippocampus, as well as the left and right MFG using the get_totals script by G. Ridgeway^2^. Masks were constructed via the wfu-pickatlas toolbox, using aal-masks for the hippocampus and 10 mm spheres around the coordinates that showed the strongest sex difference favoring women for the MFG. The extracted gray-matter volumes were analyzed using JASP 0.8.1.1 (see section “Statistical Analysis”).

For the more exploratory, whole-brain analyses, gray matter partitions were smoothed using a 12 mm Gausian kernel. The smoothed images were entered into SPM12 second level analyses. Total intracranial volume (TIV) and age were entered as covariates in all analyses. In a first step, men, naturally cycling women and OC users were compared using a one-way ANOVA design. Sexually dimorphic brain areas were identified by defining contrasts comparing men to both female groups. In addition contrasts comparing OC-users to NC women were also defined. In a second step, whole brain multiple regression designs were used, to identify areas sensitive to sex hormone levels or gender role. These whole brain regression analyses were performed separately for each group. A primary uncorrected threshold of *p* < 0.001 and a secondary cluster-level family wise error (FWE) corrected threshold of *p*_FWE_ < 0.05 were used.

### Statistical Analysis

Statistical analysis was performed using JASP 0.8.1.1. Since age differed significantly between NC and OC women, age was controlled in all analyses. For analyses of brain volumes, TIV was entered as additional covariate. Accordingly, ANCOVAs were used to compare endocrine measures, behavioral measures and brain volumes between groups, while multiple regression analyses were used to relate endocrine and behavioral measures to gray matter volumes. For group comparisons in the whole sample the omnibus test comparing all three groups (men, NC, OC) is reported in the text and pairwise comparisons are listed in Table 1. For pairwise comparisons an FDR-correction of *p*-values was used.

**TABLE 1:** Average hormone levels and masculinity/femininity self-assessment for men and women.

**Whole sample**	**men**	**women NC**	**women OC**	**Men vs. NC**		**Men vs. OC**		**NC vs. OC**	
						
	**(*n* = 89)**	**(*n* = 89)**	**(*n* = 60)**	***F***	***p***	***F***	***P***	**F**	**p**
Testosterone (pg/ml)	105.58 ± 53.24	48.43 ± 20.92	37.62 ± 18.09	**87.14**	**< 0.001**	**78.12**	**< 0.001**	**9.86**	**0.002**
Estradiol (pg/ml)	2.76 ± 1.02	3.11 ± 1.15	3.31 ± 0.94	**4.45**	**0.05**	**12.90**	**< 0.001**	0.61	0.44
Progesterone (pg/ml)	65.58 ± 55.32	136.05 ± 116.63	62.96 ± 47.82	**26.20**	**< 0.001**	0.27	0.60	**13.49**	**< 0.001**
Masculinity (self)	6.49 ± 1.16	3.83 ± 1.39	3.08 ± 1.76	**188.84**	**< 0.001**	**187.54**	**< 0.001**	**4.58**	**0.03**
*Masculinity vs. men*	5.80 ± 1.30	2.26 ± 1.10	1.88 ± 1.04	**372.11**	**< 0.001**	**333.84**	**< 0.001**	2.40	0.12
*Masculinity vs. women*	7.52 ± 1.56	5.20 ± 1.74	3.98 ± 2.11	**85.42**	**< 0.001**	**124.31**	**< 0.001**	**11.27**	**0.001**
Femininity (self)	3.60 ± 1.66	6.02 ± 1.51	6.93 ± 0.95	**263.11**	**< 0.001**	**183.05**	**< 0.001**	**16.63**	**< 0.001**
*Femininity vs. men*	4.48 ± 1.98	7.30 ± 1.70	8.05 ± 1.24	**102.27**	**< 0.001**	**139.13**	**< 0.001**	**8.65**	**0.004**
*Femininity vs. women*	2.54 ± 1.57	5.31 ± 1.71	6.33 ± 1.56	**126.75**	**< 0.001**	**192.63**	**< 0.001**	**12.38**	**< 0.001**
TIV	1582 ± 115.53	1400 ± 99.46	1400 ± 107.73	**127.53**	**< 0.001**	**98.58**	**< 0.001**	< 0.01	0.97
WM	559.9 ± 47.87	486.8 ± 47.48	479.5 ± 49.81	0.62	0.43	1.25	0.81	0.70	0.80
GM	756.0 ± 55.15	694.7 ± 52.04	688.3 ± 55.40	1.58	0.21	4.12	0.08	**9.88**	**0.006**
*HippocampusL*	4.60 ± 0.33	4.27 ± 0.38	4.13 ± 0.40	0.97	0.33	**6.82**	**0.03**	**5.18**	**0.04**
*HippocampusR*	4.15 ± 0.34	3.86 ± 0.32	3.77 ± 0.30	< 0.01	0.98	2.98	0.18	5.94	0.06
*MFG_L*	1.38 ± 0.25	1.34 ± 0.25	1.35 ± 0.28	2.72	0.30	0.21	0.65	0.35	0.55
*MFG_R*	1.24 ± 0.25	1.29 ± 0.21	1.31 ± 0.26	**19.22**	**< 0.001**	**9.69**	**0.004**	< 0.01	0.98

*Age was controlled in all comparisons. *P*-values were FDR-corrected. NC, naturally cycling. OC, oral contraceptive user. MFG, middle frontal gyrus. L, left; R, right. Significant effects are highlighted in bold font.*

Since previous work has outlined OC-dependent effects not only on gray matter volumes (Pletzer et al., 2010, 2014; Petersen et al., 2015), but also on sex hormone levels (Wiegratz et al., 2003) and on gender role (Pletzer et al., 2015b), the following analyses approach was chosen for ROI-based multiple regression analyses. In a first step it was assessed, how men differed from naturally cycling women, by accounting for biological sex in the analyses, but excluding OC-users. In a second step, naturally cycling women were compared to OC-users by accounting for OC-use in the analyses, but excluding men. Multiple regression analyses modeled age and TIV, sex hormones/gender role, and biological sex/OC-use, as well as their interactions as independent variables. If significant interactions were observed, separate partial correlations controlling for age and TIV were performed for each group to clarify.

## Results

### Endocrine Results

In the whole sample (Table 1), testosterone, progesterone and estradiol levels differed significantly between groups [T: *F*_(2,232)_ = 78.05, *p* < 0.001, η^2^ = 0.40; P: *F*_(2,227)_ = 19.26, *p* < 0.001, η^2^ = 0.15, E: *F*_(2,220)_ = 5.99, *p* = 0.005, η^2^ = 0.05]. *Post hoc* comparisons revealed that testosterone levels were significantly higher in men compared to women irrespective of their hormonal status. Progesterone and Estradiol levels were significantly higher in NC women compared to men. Testosterone and progesterone levels were significantly higher in NC women compared to OC users.

### Behavioral Results

#### Gender Role Self Assessment

In the whole sample (Table 1), significant group differences were observed in both self-rated masculinity [*F*_(2,234)_ = 119.44, *p* < 0.001, η^2^ = 0.51] and self-rated femininity [*F*_(2,234)_ = 108.23, *p* < 0.001, η^2^ = 0.48]. Men rated themselves as significantly more masculine and significantly less feminine than women irrespective if their hormonal status. Women on hormonal contraceptives rated themselves as significantly more feminine and significantly less masculine than naturally cycling women.

In men masculinity and femininity self-ratings showed a highly significant negative interrelation (*r* = −0.55, *p* < 0.001). Similarly in NC women a moderate negative association was observed between masculinity and femininity self-ratings (*r* = −0.23, *p* = 0.03). In OC women no significant association between masculinity and femininity self-ratings was observed (*r* = −0.19, *p* = 0.15). The correlation in men was significantly stronger than in the female groups (both *Z* > 2.52, both *p* < 0.012). Correlation coefficients did not differ significantly between NC and OC women (*Z* = 0.25, *p* = 0.80).

#### GERAS

Also in the GERAS, men reached significantly higher masculinity and significantly lower femininity scores compared to NC women. Differences were strongest for the interests subscale and weakest for the cognition subscale (Table 2). The masculinity and femininity subscales of the GERAS were not significantly interrelated in either men or NC women (both |*r*| < 0.15, both *p* > 0.30).

**TABLE 2:** Average hormone levels and GERAS scores for 54 men and 51 naturally cycling (NC) women.

**Subsample**	**Men (*n* = 54)**	**women NC (*n* = 51)**	***F***	***p***
Testosterone (pg/ml)	113.57 ± 62.45	43.96 ± 18.63	56.69	< 0.001
Estradiol (pg/ml)	2.40 ± 0.73	2.90 ± 1.00	8.37	0.005
Progesterone (pg/ml)	52.52 ± 53.67	153.75 ± 139.71	24.12	< 0.001
Masculinity (GERAS)	4.54 ± 0.63	4.09 ± 0.70	14.21	< 0.001
*Masculinity personality*	4.38 ± 0.63	4.14 ± 0.78	3.18	0.07
*Masculinity cognition*	4.76 ± 0.94	4.37 ± 0.96	4.29	0.04
*Masculinity interests*	4.49 ± 0.94	3.76 ± 1.17	13.92	< 0.001
Femininity (GERAS)	4.26 ± 0.59	4.92 ± 0.54	35.51	< 0.001
*Femininity personality*	4.66 ± 0.73	5.09 ± 0.74	8.53	0.004
*Femininity cognition*	4.81 ± 1.04	5.16 ± 0.68	3.93	0.05
*Femininity interests*	3.29 ± 0.82	4.53 ± 0.83	58.33	< 0.001

*Age was controlled in all comparisons.*

In men, self-rated masculinity correlated significantly with masculinity scores as assessed by the GERAS (*r* = 0.43, *p* = 0.001), while self-rated femininity did not correlate with femininity as assessed by the GERAS (*r* = −0.14, *p* = 0.33). In NC women, self-rated masculinity did not correlate with masculinity as assessed by the GERAS (*r* = 0.09, *p* = 0.54), while self-rated femininity correlated significantly with femininity as assessed by the GERAS (*r* = 0.34, *p* = 0.01). Taking into account GERAS subscales, the best predictor of men’s self-rated masculinity and women’s self-rated femininity was the personality subscale (Table 3).

**TABLE 3:** Interrelation between sex-role self-assessment and GERAS-scores.

	**Masculinity (self)**			**Femininity (self)**		
		
**Men**	***R*^2^ = 0.21, *F* = 4.62, *p* = 0.006**			***R*^2^ = 0.06, *F* = 1.18, *p* = 0.33**		

	**β**	***t***	***P***	**β**	***t***	***P***
Personality	**0.32**	**2.42**	**0.019**	0.14	0.87	0.387
Cognition	0.10	0.76	0.452	−0.27	−1.85	0.071
Interests	0.23	1.81	0.076	−0.08	−0.56	0.577

**Women**	***R* = 0.02, *F* = 0.38, *p* = 0.76**			***R* = 0.19, *F* = 3.71, *p* = 0.02**		

	**β**	***t***	***P***	**β**	***t***	***P***

Personality	0.03	0.18	0.859	**0.32**	**2.15**	**0.03**
Cognition	0.15	0.94	0.351	−0.09	−0.69	0.491
Interests	−0.04	−0.26	0.794	0.18	1.21	0.234

*Significant effects are highlighted in bold font.*

Sex hormone levels were not correlated with masculinity or femininity scores (either self-rated or assessed with the GERAS) in either men, NC-women or OC-women (all |*r*| < 0.17, all *p* > 0.20).

### Neuroimaging Results

#### Group Differences

In the whole sample (Table 1), significant group differences in TIV were observed [*F*_(2,234)_ = 80.53, *p* < 0.001, η^2^ = 0.41]. *Post hoc* comparisons revealed that TIV was significantly larger in men compared to women, but did not differ between naturally cycling women and OC users. Controlling for age and TIV, the three groups did not differ significantly in overall WM volumes [*F*_(2,233)_ = 0.89, *p* = 0.41, η^2^ = 0.002], but group differences were identified in GM volumes [*F*_(2,233)_ = 6.25, *p* = 0.002, η^2^ = 0.02]. Men and NC women did not differ in GM volumes, after age and TIV were accounted for. However, OC users had significantly smaller overall GM volumes than NC women.

In the ROI analyses, significant group differences were observed in the left hippocampus [*F*_(2,233)_ = 4.00, *p* = 0.02, η^2^ = 0.03] and the right MFG [*F*_(2,233)_ = 11.74, *p* < 0.001, η^2^ = 0.07]. Pairwise comparisons revealed that OC users showed significantly smaller gray matter volumes in the left hippocampus than NC women. Men showed significantly smaller volume in the right MFG than women irrespective of their hormonal status. No significant group differences were observed in the right hippocampus [*F*_(2,233)_ = 2.66, *p* = 0.07, η^2^ = 0.02] and the left MFG [*F*_(2,233)_ = 1.28, *p* = 0.28, η^2^ = 0.01].

**FIGURE 1:**
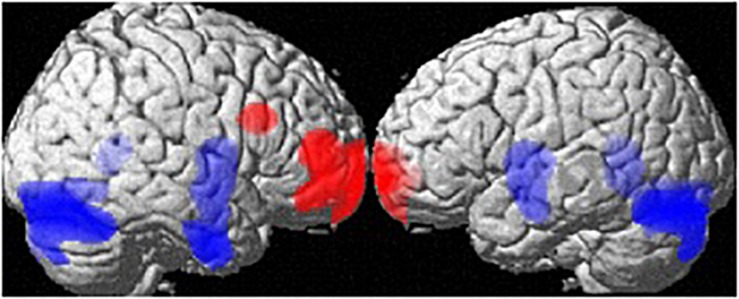
Sex differences in regional gray matter volumes. Areas with larger regional volumes in men are depicted in blue (Cerebellum, Hippocampus/Parahippocampus, Amygdala, Putamen). Areas with larger regional volumes in women are depicted in red (frontal pole, middle/inferior frontal gyrus).

At the whole-brain level, regional volume differences between men and women (both groups) are depicted in Figure 1. Controlling for age and TIV, men showed larger GM-volumes than women in the cerebellum ([34, −78, −20], 16405 voxels, *T* = 6.86, *p*_FWE_ < 0.001) and a large cluster spanning the bilateral putamen, hippocampi, parahippocampi and amygdalae [(26, −3, −9), 7319 voxels, *T* = 5.00, *p*_FWE_ = 0.005]. Women showed larger GM volumes than men in the frontal pole [(−18, 68, −4), 4241 voxels, *T* = 5.01, *p*_FWE_ = 0.005], right MFG [(36,18,27), 627 voxels, *T* = 5.01, *p*_FWE_ = 0.004; Table 1] and right IFG [(46,50,10), 2491 voxels, *T* = 4.95, *p*_FWE_ = 0.006].

**FIGURE 2:**
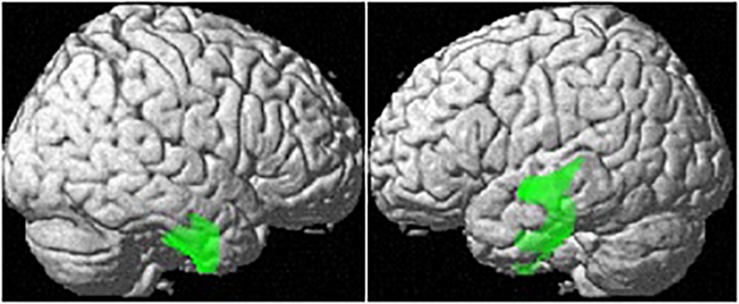
Differences in regional GM volumes between naturally cycling women and OC users. Areas with smaller regional volumes in OC users are depicted in green.

Controlling for age and TIV, OC users showed significantly smaller regional GM-volumes than naturally cycling women in the right parahippocampal/fusiform gyrus [(28, −14, −42), 1882 voxels, *T* = 5.29, *p*_FWE_ = 0.001; Figure 2].

#### Sex Hormones and GM-Volumes

In men and NC women, sex hormones were not related to TIV, overall GM or WM volumes (all |*r*| < 0.17, all *p* > 0.11). In OC users, estradiol levels were significantly related to TIV and overall GM and WM volumes (all *r* > 0.25, all *p* < 0.05; results not shown), but there was no association between testosterone or progesterone levels and TIV/GM/WM (all |*r*| < 0.20, all *p* > 0.13). The higher the estradiol levels of OC users, the larger were their brains.

**FIGURE 3:**
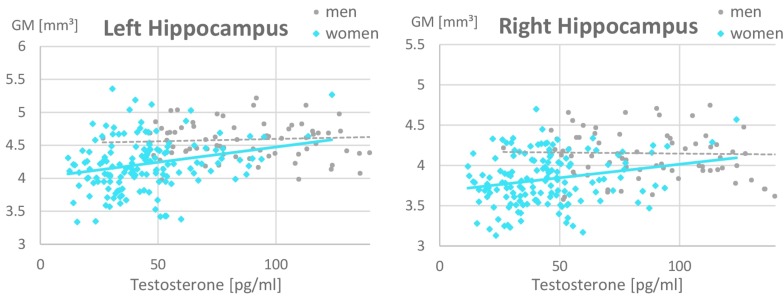
Relationship of testosterone to hippocampal gray matter volumes. A positive relationship was observed in women (left: *r* = 0.26, *p* = 0.02; right: *r* = 0.16, *p* = 0.15) but not in men (left: *r* = 0.07, *p* = 0.53; right: *r* = –0.14, *p* = 0.19). Women with higher Testosterone levels, showed larger hippocampal gray matter volumes. This association was irrespective of oral contraceptive (OC) use, i.e., both naturally cycling women and OC users are included in the female data depicted. For illustrative purposes, the x-scale was cut at 150 pg/ml of Testosterone. Note that male values reached up to 350 pg/ml.

In the ROI-based analyses of sex hormones, significant sex × testosterone interactions were identified in the hippocampi, which are attributable to the fact that testosterone related more positively to hippocampal volumes in women (left: *r* = 0.26, *p* = 0.02; right: *r* = 0.16, *p* = 0.15) than in men (left: *r* = 0.07, *p* = 0.53; right: *r* = −0.14, *p* = 0.19; Figure 3 and Table 4). This association did not differ between OC-users and NC women (Table 5).

**TABLE 4:** Relationship of sex hormones to gray matter volumes in the hippocampus and middle frontal gyrus (MFG) while controlling for biological sex.

	**HippocampusL**		**HippocampusR**		**MFG_L**		**MFG_R**	
				
	***b***	***t***	***b***	***t***	***b***	***t***	***b***	***T***
Sex	0.10	0.97	0.02	0.21	**0.24**	**1.99^∗^**	**0.30**	**2.55^∗∗^**
Age	–0.02	–0.33	–0.05	–0.73	**–0.19**	**–2.54^∗^**	**-0.21**	**–2.81^∗∗^**
**TIV**	**0.55**	**6.56^∗∗∗^**	**0.64**	**7.69^∗∗∗^**	**0.39**	**3.92^∗∗∗^**	**0.51**	**5.23^∗∗∗^**
Estradiol	–0.11	–1.56	–0.10	–1.43	–0.03	–0.34	–0.07	–0.88
Progesterone	–0.01	–0.15	0.10	1.07	–0.03	–0.22	0.16	1.44
Testosterone	**0.37**	**2.88^∗∗^**	0.12	0.92	0.16	1.07	–0.18	–1.19
Sex × Estradiol	–0.01	–0.09	0.03	0.37	–0.02	–0.26	0.06	–0.72
Sex × Prog	0.05	0.58	–0.06	–0.66	–0.13	–1.19	–0.14	–1.35
Sex × Test	**0.26**	**2.44^∗^**	**0.22**	**2.05^∗^**	0.19	1.50	–0.10	–0.83

*MFG = middle frontal gyrus. L, left; R, right. ^∗^*p* < 0.05, ^∗∗^*p* < 0.01, and ^∗∗∗^*p* < 0.001. Significant effects are highlighted in bold font.*

**TABLE 5:** Relationship of sex hormones to gray matter volumes in women, while controlling for OC-use.

	**HippocampusL**		**HippocampusR**		**MFG_L**		**MFG_R**	
				
	***b***	***t***	***b***	***t***	***b***	***t***	***b***	***T***
OC-use	–0.20	–1.48	–0.17	–1.35	**–0.46**	**–3.09^∗∗^**	–0.10	–0.66
Age	–0.01	–0.17	–0.07	–0.91	**–0.18**	**–2.05^∗^**	–0.14	–1.55
**TIV**	**0.51**	**6.97^∗∗∗^**	**0.60**	**8.50^∗∗∗^**	**0.24**	**2.93^∗∗^**	**0.37**	**4.48^∗∗∗^**
Estradiol	–0.10	–1.03	–0.08	–0.85	–0.05	–0.46	–0.004	–0.04
Progesterone	0.04	0.49	0.05	0.63	–0.16	–1.60	–0.002	–0.02
Testosterone	**0.25**	**2.40^∗^**	0.17	1.68	0.16	1.37	–0.13	–1.10
OC × Estradiol	0.02	0.24	0.02	0.27	0.15	1.49	0.05	0.46
OC × Prog	–0.01	–0.13	–0.05	–0.54	0.15	1.44	–0.01	–0.13
OC × Test	–0.15	–0.93	–0.09	–0.57	**–0.51**	**–2.90^∗∗^**	–0.02	–0.09

*MFG = middle frontal gyrus. L = left, R = right. ^∗^*p* < 0.05, ^∗∗^*p* < 0.01, ^∗∗∗^*p* < 0.001. Significant effects are highlighted in bold font.*

For the left MFG, a significant interaction between OC-use and testosterone was observed (Table 5). This interaction resulted from a negative association to testosterone in OC women (*r* = −0.27, *p* = 0.04), but non-significant association in NC women (*r* = 0.13, *p* = 0.25).

No additional associations to sex hormones were observed in whole-brain analyses.

#### Gender Role and GM-Volumes

In men and NC women, neither self-rated nor GERAS-masculinity or femininity were related to total TIV, GM or WM volumes (all |*r*| < 0.15, all *p* > 0.17). In OC users, self-rated femininity was negatively related to TIV (*r* = −0.25, *p* = 0.05; results not shown).

Neither Self-rated nor GERAS masculinity or femininity were related to GM-volumes in any ROI and there were no differences in these associations depending on biological sex or OC-use (all |*b*| < 0.26, all |*t*| < 1.98, all *p* > 0.05).

**FIGURE 4:**
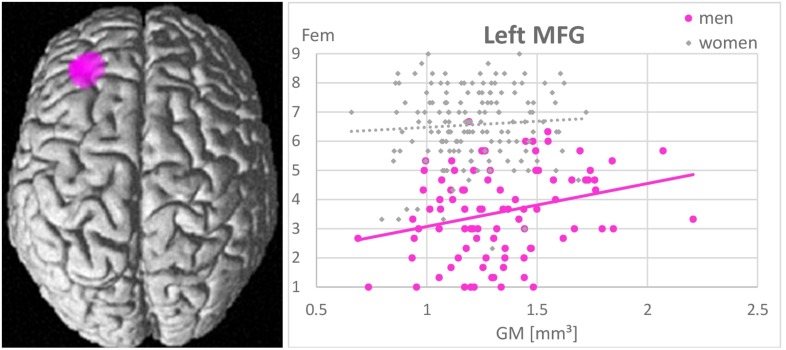
Association between femininity (Fem) and gray matter volumes in the left middle frontal gyrus (MFG). A positive relationship was observed in men (*r* = 0.44, *p* < 0.001), but not in women (*r* = 0.07, *p* = 0.42). The larger the left MFG, the more feminine did men consider themselves.

Whole-brain analyses revealed no associations between gender role as assessed by the GERAS and GM volumes in any brain area. In men larger GM volumes in the left MFG were significantly positively related to higher femininity ratings [(−32, 36, 22), *k* = 721 voxels, *T* = 5.01, *p*_FWE_ = 0.015; Figure 4]. The more GM in the left MFG, the more feminine did men consider themselves. Masculinity self-ratings were not related to GM volumes in any area. In NC-women and OC-users masculinity and femininity self-ratings were not related to GM volumes in any area.

## Discussion

The present study set out to (i) investigate the relationship of circulating sex hormone levels and gender role to gray matter volumes in sexually dimorphic brain areas and explore, whether these relationships are modulated by (ii) biological sex and (iii) OC-use. Indeed, a variety of associations between sex hormones and gender role to gray matter volumes were observed, that were dependent on either biological sex or OC-use. The fact that gender role and sex hormones showed no significant interrelation in this sample underlines the view of gender role as a social construct and provides the opportunity to study their influence on gray matter volumes independently.

In accordance with our hypothesis, testosterone related positively to gray matter volumes in the hippocampi (Figure 3), i.e., brain areas showing a sexual dimorphism favoring men. These findings are in good accordance with studies in transsexuals, demonstrating that cross-sex hormone treatment alters brain structure toward the proportions of the aspired sex (Hulshoff Pol et al., 2006; see Guillamon et al., 2016 for a review). Furthermore, they are in good accordance with animal studies, demonstrating a testosterone-dependent increase in synaptic spine density in the hippocampus (e.g., Leranth et al., 2003). The association was significantly stronger in women compared to men, but did not differ significantly between NC women and OC-users, even though the strongest associations were observed in NC women. This observation is consistent with the animal literature showing testosterone actions on hippocampal spine density via local conversion to estradiol also only in females (see Atwi et al., 2016 for a review).

Furthermore, in line with our hypothesis, a positive association between self-rated femininity and gray matter volumes in the left MFG, i.e., a brain area typically larger in women, was observed in men. Men, who perceive themselves as more feminine, show larger left MFG volumes. This is in line with a previous study reporting larger cortical thickness in the left MFG of untreated male-to-female transsexuals compared to men (Luders et al., 2012). This association probably reflects an important role of the MFG in personality traits and other characteristics, typically considered as feminine. Results of the present study support the assumption that gender role self-concepts are largely driven by personality (compare Table 3). As an example, conscientiousness (Schmitt et al., 2008), which is typically higher in women, was shown to relate to gray matter volumes in the lateral prefrontal cortex (DeYoung et al., 2010).

It is an interesting observation that female brain structure relates more strongly to sex hormone levels than male brain structure, while male brain structure relates more strongly to gender roles than female brain structure. While several associations between gray matter volumes and testosterone were observed in women, no significant association to sex hormones were observed in men. Based on this observation it can be speculated whether the female brain is more susceptible to sex hormone influences, which is plausible given the continuous plasticity required to respond to hormonal fluctuations along the menstrual cycle (e.g., Pletzer et al., 2018; see Sundström Poromaa and Gingnell, 2014 for a review) or during other hormonal transition periods (e.g., pregnancy, menopause, see Barth et al., 2015 for a review). Notably, however, no associations to estradiol or progesterone were observed in women, even though such influences have been demonstrated in within-subjects designs in women (Barth et al., 2016; Pletzer et al., 2018). It is possible that gray matter volumes are not so much dependent on the absolute circulating hormone level, as measured in the present study, but respond to sudden changes in hormone levels as can only be assessed in within-subject designs.

Vice versa, no association between gender role and brain structure was observed in women. This finding is in line with previous research on transsexual and homosexual participants. Altered regional brain morphology was only observed in untreated male-to-female transsexuals and male homosexual participants (Luders et al., 2012; Abé et al., 2014) not in female-to-male transsexuals or female homosexual participants (Guillamon et al., 2016; Manzouri and Savic, 2018). Furthermore, a stronger association of personality traits to brain structure in men compared to women has also been previously reported (Nostro et al., 2016). One research question that arises from this observation is whether the stability of personality traits or gender role constructs differs between men and women. If women’s gender role self-concept is more flexible over time, a relationship to brain structure is not to be expected. While this has hardly been assessed for personality or gender identity, a higher flexibility in women has been reported regarding sexual orientation (Kinnish et al., 2005), which may explain the lack of brain structural differences between homosexual and heterosexual women (Manzouri and Savic, 2018).

However, apart from the relationship of self-reported femininity to the left MFG volumes in men, no association between masculinity or femininity (neither self-rated, nor questionnaire-based) and brain structure was observed. Again, this is in line with previous literature on transsexual individuals reporting that before sex hormone treatment their brain morphology largely corresponds to their natal sex (Guillamon et al., 2016). While personality traits have been successfully related to brain morphology, it is important to keep in mind, that the gender role self-concept develops at the interplay between an individual’s traits, abilities and interests on the one hand and social norms on the other hand. While some traits may relate to different brain structures, the same traits and abilities may result in different perceptions of masculinity/femininity in different cultural contexts. Furthermore – as the factorial structure of the GERAS shows – gender role is a multi-facetted construct spanning a variety of traits, abilities and interests, which cannot all be pinpointed to the same brain area. It is, however, possible, that in the left MFG several of the traits contributing to femininity intersect. The fact that men tend to show stronger lateralization of brain functions may also have contributed to this finding (e.g., Hausmann and Güntürkün, 1999, 2000).

Finally, some important differences between naturally cycling women and OC-users have been identified. In interpreting these differences it is important to keep in mind, that the results reported here represent between-group comparisons. It is thus possible that the groups of OC-users and NC-women differ for other reasons than their OC-use. First, OC users show significantly lower testosterone and progesterone levels than NC women, which is probably a result of the downregulation of the HPG-axis by synthetic steroids (Wiegratz et al., 2003). Estradiol levels did not differ significantly between OC-user and NC women. This may be due to the fact that none of the NC-women tested in the present study were in the pre-ovulatory phase, when estradiol levels peak, but may also be the result of some cross-reactivity between the synthetic ethinyl-estradiol and the antibodies used for estradiol assessment. Second, the finding that OC-users rate themselves as more feminine and significantly less masculine compared to naturally cycling women was replicated (Pletzer et al., 2015b). This observation does not necessarily imply a hormonal modulation of gender role. There are various non-hormonal reasons why women on OCs may perceive themselves as more feminine. On the one hand, the daily intake of a pill controlling one’s reproductive functions may act as a constant reminder of one’s own femininity. It is also possible, that the heightened femininity is not a result of the OC-use, but that women who consider themselves more feminine are more likely to choose OCs as a contraceptive method. This is probably related to the fact that a majority of women start OC-use when entering a long-term relationship. Accordingly, the increased femininity may be a result or a pre-requisite of OC-users different relationship status. Note, however, that relationship status was not assessed in the present study.

Furthermore, several differences in brain volume results between OC-users and NC women were observed. The fact that OC-users show larger TIV is likely attributable to chance in sampling, which represents another issue in between-group comparisons. More importantly, OC-users show smaller regional GM volumes than NC women in the hippocampi and parahippocampal gyri. These results are in contrast to previous studies demonstrating larger gray matter volumes of OC-users compared to non-users in the hippocampus, parahippocampus and fusiform gyri (Pletzer et al., 2010, 2015a; De Bondt et al., 2013). These inconsistencies between studies highlight the importance of longitudinal study designs to disentangle effects of OC-use from other variables that might differentiate OC-users and NC-women in cross-sectional designs. The inconsistencies may be a result of different actions of the various progestin compounds contained in different OCs. The majority of OC-users in the present study used OCs containing androgenic progestins, i.e., progestins that are derived from 19-nortestosterone and thus able to bind to testosterone receptors. Previous findings of increased parahippocampal/fusiform volumes were observed in users of anti-androgenic progestins (Pletzer et al., 2015a). A reduction of GM-volumes, has previously been reported for androgenic progestins, albeit in the MFG (Pletzer et al., 2015a). Comparably, in the present study, OC-users show smaller left MFG-volumes with higher testosterone levels.

In summary, our study corroborates findings of activational effects of sex hormones on brain morphology in adults, demonstrating that – at least in women – testosterone promotes a more male-like brain morphology and estradiol a more female-like brain morphology. In addition our study also demonstrates for the first time an association between a more feminine gender role and a more female-like brain morphology in men. Finally our study identifies differences in gender role and gray matter volumes between OC-users and naturally cycling women.

## Data Availability

The datasets generated for this study are available on request to the corresponding author.

## Author Contributions

BP designed the study, analyzed the data, and wrote the manuscript.

## Conflict of Interest Statement

The author declares that the research was conducted in the absence of any commercial or financial relationships that could be construed as a potential conflict of interest.

## Footnotes

1 http://www.demeditec.com

2 http://www0.cs.ucl.ac.uk/staff/gridgway/vbm/get_totals.m

## References

[B1] Abé C. Johansson E. Allzén E. Savic I. (2014). Sexual orientation related differences in cortical thickness in male individuals. *PLoS One* 9:e114721. 10.1371/journal.pone.0114721 25479554 PMC4257718

[B2] Andreano J. M. Cahill L. (2009). Sex influences on the neurobiology of learning and memory. *Learn. Mem.* 16 248–266. 10.1101/lm.918309 19318467

[B3] Atwi S. McMahon D. Scharfman H. MacLusky N. J. (2016). Androgen modulation of hippocampal structure and function. *Neuroscientist* 22 46–60. 10.1177/1073858414558065 25416742 PMC5002217

[B4] Barth C. Steele C. J. Mueller K. Rekkas V. P. Arélin K. Pampel A. (2016). In-vivo dynamics of the human hippocampus across the menstrual cycle. *Sci. Rep.* 6:32833. 10.1038/srep32833 27713470 PMC5054394

[B5] Barth C. Villringer A. Sacher J. (2015). Sex hormones affect neurotransmitters and shape the adult female brain during hormonal transition periods. *Front. Neurosci.* 9:37. 10.3389/fnins.2015.00037 25750611 PMC4335177

[B6] Bem S. L. (1974). The measurement of psychological androgyny. *J. Consult. Clin. Psychol.* 42:155. 4823550

[B7] Bem S. L. (1981). *Bem Sex-Role Inventory Mental Measurements Yearbook with Tests in Print.* Ipswich: EBSCO.

[B8] Chapple C. L. Vaske J. Hope T. L. (2010). Sex differences in the causes of self-control: an examination of mediation, moderation, and gendered etiologies. *J. Crim. Just.* 38 1122–1131. 10.1016/j.jcrimjus.2010.08.004

[B9] Cosgrove K. P. Mazure C. M. Staley J. K. (2007). Evolving knowledge of sex differences in brain structure, function, and chemistry. *Biol. Psychiatry* 62 847–855. 10.1016/j.biopsych.2007.03.001 17544382 PMC2711771

[B10] Courvoisier D. S. Renaud O. Geiser C. Paschke K. Gaudy K. Jordan K. (2013). Sex hormones and mental rotation: an intensive longitudinal investigation. *Horm. Behav.* 63 345–351. 10.1016/j.yhbeh.2012.12.007 23261859

[B11] De Bondt T. Jacquemyn Y. Van Hecke W. Sijbers J. Sunaert S. Parizel P. M. (2013). Regional grey matter volume differences and sex-hormone correlations as a function of menstrual cycle phase and hormonal contraceptives use. *Brain Res.* 1530 22–31. 10.1016/j.brainres.2013.07.034 23892107

[B12] DeYoung C. G. Hirsh J. B. Shane M. S. Papademetris X. Rajeevan N. Grey J. R. (2010). Testing predictions from personality neuroscience: Brain structure and the big five. *Psychol. Sci.* 21 820–828. 10.1177/0956797610370159 20435951 PMC3049165

[B13] Driscoll I. Hamilton D. A. Yeo R. A. Brooks W. M. Sutherland R. J. (2005). Virtual navigation in humans: the impact of age, sex, and hormones on place learning. *Horm. Behav.* 47 326–335. 10.1016/j.yhbeh.2004.11.013 15708762

[B14] Eagly A. H. Koenig A. M. (2006). “Social role theory of sex differences and similarities: implication for prosocial behavior,” in *Sex Differences and Similarities in Communication*, eds Dindia K. Canary D. J. (Mahwah, NJ: Lawrence Erlbaum Associates Publishers), 161–177.

[B15] Gruber F. Scherndl T. Ortner T. Pletzer B. (in press). Psychometric properties of the multifaceted gender-related attributes survey (GERAS). *Eur. J. Psychol. Assess.* 10.1027/1015-5759/a000528 PMC7116055 32913384

[B16] Guillamon A. Junque C. Gómez-Gil E. (2016). A review of the status of brain structure research in transsexualism. *Arch. Sex. Behav.* 45 1615–1648. 10.1007/s10508-016-0768-5 27255307 PMC4987404

[B17] Gur R. E. Gur R. C. (2016). Sex differences in brain and behavior in adolescence: findings from the Philadelphia Neurodevelopmental Cohort. *Neurosci. Biobehav. Rev.* 70 159–170. 10.1016/j.neubiorev.2016.07.035 27498084 PMC5098398

[B18] Hao J. Rapp P. R. Leffler A. E. Leffler S. R. Janssen W. G. Lou W. (2006). Estrogen alters spine number and morphology in prefrontal cortex of aged female rhesus monkeys. *J. Neurosci.* 26 2571–2578. 10.1523/jneurosci.3440-05.2006 16510735 PMC6793646

[B19] Hausmann M. Güntürkün O. (1999). Sex differences in functional cerebral asymmetries in a repeated measures design. *Brain Cogn.* 41 263–275. 10.1006/brcg.1999.1126 10585238

[B20] Hausmann M. Güntürkün O. (2000). Steroid fluctuations modify functional cerebral asymmetries: the hypothesis of progesterone-mediated interhemispheric decoupling. *Neuropsychologia* 38 1362–1374. 10.1016/s0028-3932(00)00045-2 10869579

[B21] Hausmann M. Schoofs D. Rosenthal H. E. Jordan K. (2009). Interactive effects of sex hormones and gender stereotypes on cognitive sex differences—A psychobiosocial approach. *Psychoneuroendocrinology* 34 389–401. 10.1016/j.psyneuen.2008.09.019 18992993

[B22] Hooven C. K. Chabris C. F. Ellison P. T. Kosslyn S. M. (2004). The relationship of male testosterone to components of mental rotation. *Neuropsychologia* 42 782–790. 10.1016/j.neuropsychologia.2003.11.012 15037056

[B23] Hornung J. Smith E. Junger J. Pauly K. Habel U. Derntl B. (2019). Exploring sex differences in the neural correlates of self-and other-referential gender stereotyping. *Front. Behav. Neurosci.* 13:31. 10.3389/fnbeh.2019.00031 30833893 PMC6387933

[B24] Hosseini-Kamkar N. Morton B. J. (2014). Sex differences in self-regulation: an evolutionary perspective. *Front. Neurosci.* 8:233. 10.3389/fnins.2014.00233 25140126 PMC4121536

[B25] Hulshoff Pol H. E. H. Cohen-Kettenis P. T. Van Haren N. E. Peper J. S. Brans R. G. Cahn W. (2006). Changing your sex changes your brain: influences of testosterone and estrogen on adult human brain structure. *Eur. J. Endocrinol.* 155 (Suppl. 1), S107–S114.

[B26] Jäncke L. Mérillat S. Liem F. Hänggi J. (2015). Brain size, sex, and the aging brain. *Hum. Brain Map.* 36 150–169. 10.1002/hbm.22619 PMC6869393 25161056

[B27] Kelly S. J. Ostrowski N. L. Wilson M. A. (1999). Gender differences in brain and behavior: hormonal and neural bases. *Pharmacol. Biochem. Behav.* 64 655–664. 10593187 10.1016/s0091-3057(99)00167-7

[B28] Kinnish K. K. Strassberg D. S. Turner C. W. (2005). Sex differences in the flexibility of sexual orientation: a multidimensional retrospective assessment. *Arch. Sex. Behav.* 34 173–183. 10.1007/s10508-005-1795-9 15803251

[B29] Leranth C. Petnehazy O. MacLusky N. J. (2003). Gonadal hormones affect spine synaptic density in the CA1 hippocampal subfield of male rats. *J. Neurosci.* 23 1588–1592. 10.1523/jneurosci.23-05-01588.2003 12629162 PMC6741990

[B30] Levine S. C. Foley A. Lourenco S. Ehrlich S. Ratliff K. (2016). Sex differences in spatial cognition: advancing the conversation. *Wiley Interdiscipl. Rev. Cogn. Sci.* 7 127–155. 10.1002/wcs.1380 26825049

[B31] Lisofsky N. Mårtensson J. Eckert A. Lindenberger U. Gallinat J. Kühn S. (2015). Hippocampal volume and functional connectivity changes during the female menstrual cycle. *Neuroimage* 118 154–162. 10.1016/j.neuroimage.2015.06.012 26057590

[B32] Luders E. Sánchez F. J. Tosun D. Shattuck D. W. Gaser C. Vilain E. (2012). Increased cortical thickness in male-to-female transsexualism. *J. Behav. Brain Sci.* 2:357. 10.4236/jbbs.2012.23040 23724358 PMC3665407

[B33] Manzouri A. Savic I. (2018). Cerebral sex dimorphism and sexual orientation. *Hum. Brain Mapp.* 39 1175–1186. 10.1002/hbm.23908 29227002 PMC6866632

[B34] Nash S. C. (1979). “Sex role as mediator of intellectual functioning,” in *Sex-Related Differences in Cognitive Functioning: Developmental Issues*, eds Wittig M. A. Petersen A. C. (New York, NY: Academic), 263–302.

[B35] Nielsen S. E. Ertman N. Lakhani Y. S. Cahill L. (2011). Hormonal contraception usage is associated with altered memory for an emotional story. *Neurobiol. Learn. Mem.* 96 378–384. 10.1016/j.nlm.2011.06.013 21740976 PMC3148336

[B36] Nostro A. D. Müller V. I. Reid A. T. Eickhoff S. B. (2016). Correlations between personality and brain structure: a crucial role of gender. *Cereb. Cortex* 27 3698–3712. 10.1093/cercor/bhw191 27390020 PMC6059198

[B37] Petersen N. Touroutoglou A. Andreano J. M. Cahill L. (2015). Oral contraceptive pill use is associated with localized decreases in cortical thickness. *Hum. Brain Mapp.* 36 2644–2654. 10.1002/hbm.22797 25832993 PMC4478200

[B38] Pletzer B. (2016). Sex differences in number processing: differential systems for subtraction and multiplication were confirmed in men, but not in women. *Sci. Rep*. 6:39064 10.1038/srep39064 PMC5155285 27966612

[B39] Pletzer B. Harris T. (2018). Sex hormones modulate the relationship between global advantage, lateralization, and interhemispheric connectivity in a navon paradigm. *Brain Connect*. 8, 106–118 10.1089/brain.2017.0504 PMC5865260 29226703

[B40] Pletzer B. Harris T. Hidalgo-Lopez E. (2018). Subcortical structural changes along the menstrual cycle: beyond the hippocampus. *Sci. Rep.* 8:16042. 10.1038/s41598-018-34247-4 30375425 PMC6207699

[B41] Pletzer B. Kronbichler M. Aichhorn M. Bergmann J. Ladurner G. Kerschbaum H. H. (2010). Menstrual cycle and hormonal contraceptive use modulate human brain structure. *Brain Res.* 1348 55–62. 10.1016/j.brainres.2010.06.019 20550945

[B42] Pletzer B. Kronbichler M. Nuerk H. C. Kerschbaum H. (2014). Hormonal contraceptives masculinize brain activation patterns in the absence of behavioral changes in two numerical tasks. *Brain Res.* 1543 128–142. 10.1016/j.brainres.2013.11.007 24231554

[B43] Pletzer B. Kronbichler M. Kerschbaum H. (2015a). Differential effects of androgenic and anti-androgenic progestins on fusiform and frontal grey matter volume and face recognition performance. *Brain Res.* 1596 108–115. 10.1016/j.brainres.2014.11.025 25446458

[B44] Pletzer B. Ortner T. M. (2016). Neuroimaging supports behavioral personality assessment: Overlapping activations during reflective and impulsive risk taking. *Biol. Psychol*. 119, 46–53 10.1016/j.biopsycho.2016.06.012 27373370

[B45] Pletzer B. Petasis O. Ortner T. Cahill L. (2015b). Interactive effects of culture and sex hormones on the sex role self-concept. *Front. Neurosci.* 9:240. 10.3389/fnins.2015.00240 26236181 PMC4500910

[B46] Ponseti J. Siebner H. R. Klöppel S. Wolff S. Granert O. Jansen O. (2007). Homosexual women have less grey matter in perirhinal cortex than heterosexual women. *PLoS One* 2:e762. 10.1371/journal.pone.0000762 17712410 PMC1942120

[B47] Protopopescu X. Butler T. Pan H. Root J. Altemus M. Polanecsky M. (2008). Hippocampal structural changes across the menstrual cycle. *Hippocampus* 18 985–988. 10.1002/hipo.20468 18767068

[B48] Reilly D. Neumann D. L. (2013). Gender-role differences in spatial ability: a meta-analytic review. *Sex Roles* 68 521–535. 10.1007/s11199-013-0269-0

[B49] Riccelli R. Toschi N. Nigro S. Terracciano A. Passamonti L. (2017). Surface-based morphometry reveals the neuroanatomical basis of the five-factor model of personality. *Soc. Cogn. Affect. Neurosci.* 12 671–684. 10.1093/scan/nsw175 28122961 PMC5390726

[B50] Ritchie S. J. Cox S. R. Shen X. Lombardo M. V. Reus L. M. Alloza C. (2018). Sex differences in the adult human brain: evidence from 5216 UK Biobank participants. *Cereb. Cortex* 28 2959–2975. 10.1093/cercor/bhy109 29771288 PMC6041980

[B51] Ruigrok A. N. Salimi-Khorshidi G. Lai M. C. Baron-Cohen S. Lombardo M. V. Tait R. J. (2014). A meta-analysis of sex differences in human brain structure. *Neurosci. Biobehav. Rev.* 39 34–50. 10.1016/j.neubiorev.2013.12.004 24374381 PMC3969295

[B52] Schmitt D. P. Realo A. Voracek M. Allik J. (2008). Why can’t a man be more like a woman? sex differences in big five personality traits across 55 cultures. *J. Personal. Soc. Psychol.* 94:168. 10.1037/0022-3514.94.1.168 18179326

[B53] Spence J. T. Helmreich R. Stapp J. (1975). Ratings of self and peers on sex role attributes and their relation to self-esteem and conceptions of masculinity and femininity. *J. Personal. Soc. Psychol.* 32:29. 10.1037/h0076857 1206468

[B54] Sundström Poromaa I. Gingnell M. (2014). Menstrual cycle influence on cognitive function and emotion processing—from a reproductive perspective. *Front. Neurosci.* 8:380. 10.3389/fnins.2014.00380 25505380 PMC4241821

[B55] Vafaei A. Ahmed T. Freire A. D. N. F. Zunzunegui M. V. Guerra R. O. (2016). Depression, sex and gender roles in older adult populations: the international mobility in aging study (IMIAS). *PLoS One* 11:e0146867. 10.1371/journal.pone.0146867 26771828 PMC4714885

[B56] Wiegratz I. Kutschera E. Lee J. H. Moore C. Mellinger U. Winkler U. H. (2003). Effect of four different oral contraceptives on various sex hormones and serum-binding globulins. *Contraception* 67 25–32. 10.1016/s0010-7824(02)00436-5 12521654

[B57] Woolley C. S. McEwen B. S. (1993). Roles of estradiol and progesterone in regulation of hippocampal dendritic spine density during the estrous cycle in the rat. *J. Comp. Neurol.* 336 293–306. 10.1002/cne.903360210 8245220

[B58] Woolley C. S. McEwen B. S. (1994). Estradiol regulates hippocampal dendritic spine density via an N-methyl-D-aspartate receptor-dependent mechanism. *J. Neurosci.* 14 7680–7687. 10.1523/jneurosci.14-12-07680.1994 7996203 PMC6576901

